# American Association of Obstetricians and Gynecologists

**Published:** 1904-10

**Authors:** 


### American Association of Obstetricians and Gynecologists.

THE seventeenth annual meeting of this association, held at Saint Louis, September 13, 14, 15 and 16, 1904, under the presidency of Dr. Walter Blackburn Dorsett, of that city, in some respects was noteworthy and is destined to become famous in the history of the association.

It is a dangerous experiment to hold a scientific medical meeting within the shadow of such an attraction as the great World's Fair, now holding forth at Saint Louis. The temptation to desert the sessions for the exhibition with its numberless allurements, is a factor to be reckoned with in appointing such a meeting. In this instance it was met by holding only morning sessions and extending them one day beyond the usual custom, giving four mornings to the entire work. This plan proved a satisfactory solution of the difficulty, the house being full for four hours every morning, the afternoons and evenings being given over to the exposition for all who so desired. This has been a hard working association for sixteen years and deserved the little recreation that the plan carried out at Saint Louis afforded.

The work done at this meeting will take rank along with that heretofore consummated by the association, which even its critics acknowledge to be not second to that of any other similar body. In spite of great difficulties, in the face of contemptuous opposition, notwithstanding desperate attempts to lure some of its prominent members away from its guild into a rival society, (happily without success), without congresses to lean upon—even despite them—this association has gradually risen from obscurity to fame, until now it occupies a position quite in the forefront of the splendid group of American special societies.

At the Saint Louis meeting the attendance was large, the papers were of a high order, while the discussions were crisp, snappy, logical, even in some instances conclusive, and always full of interest. The social entertainments were singularly felicitous, in which were included an automobile excursion about the city for the ladies, under the direction of the committee of arrangements, and the reception given at the Missouri State building within the exposition grounds, by the Saint Louis Obstetrical and Gynecological Society, led by its accomplished president, Dr. L. E. Newman. The banquet at The Monticello, Thursday evening, was unusually well-appointed, the speeches being of a quality beyond the ordinary pitch at such dinners. Every speaker was original, witty, even brilliant in his response to the demands of the toastmaster. The vocal singing that interspersed the speaking was effective and lent zest to an already sparkling, charming event.

The Saint Louis meeting will be remembered long by those who participated and the distinguished president, together with his Saint Louis colleagues, merited and received the thanks of the association for providing so liberally for the mental and physical entertainment of the visitors and members.

The association voted to hold its next meeting at New York in September, 1905, and elected officers for the eighteenth year as follows: president, Howard W. Longyear, Detroit; vice-presidents, D. Tod Gilliam, Columbus, and John Young Brown, Saint Louis; secretary, William Warren Potter, Buffalo; treasurer, Xavier O. Werder, Pittsburg; executive councillors (to fill vacancies) James F. W. Ross, Toronto, and Walter B. Dorsett, Saint Louis.

